# Nurturing Holistic Development of University Students in Hong Kong: Where Are We and Where Should We Go?

**DOI:** 10.1100/tsw.2010.62

**Published:** 2010-04-01

**Authors:** Daniel T. L. Shek

**Affiliations:** ^1^ Department of Applied Social Sciences, Hong Kong Polytechnic University, Hong Kong; ^2^ Department of Sociology, East China Normal University, Shanghai, China; ^3^ Public Policy Research Institute, Hong Kong Polytechnic University, Hong Kong; ^4^ Kiang Wu Nursing College of Macau, Macao

**Keywords:** positive youth development, general education, holistic development, university students

## Abstract

With reference to the mental health and developmental problems among university students, there is a need to review the university's role in nurturing holistic development of students. This paper explores the question of how holistic development of university students in Hong Kong can be promoted. Based on the positive youth development approach, it is argued that promotion of intrapersonal competencies, interpersonal relationship skills, civic responsibilities, and citizenship among university students is an important strategy to facilitate holistic development of young people in Hong Kong. Two general education or freshman seminar courses that focus on the cultivation of intrapersonal competencies, interpersonal relationship skills, civic responsibilities, and sense of citizenship among university students in Hong Kong are proposed.

### INTRODUCTION

Higher education in Hong Kong is in the process of transformation. In 2012, the present 3-year university system modeled after the British model will be changed to a 4-year university system that is commonly adopted in North America and China. Obviously, one basic question that academics and university senior management should seriously consider is how university education can nurture university students who are young people in Hong Kong. To formulate a strategy to promote holistic development of university students, we must understand the psychosocial needs and developmental profiles of young people in Hong Kong.

There are several observations regarding adolescent development in Hong Kong. First, research and statistics show that there are several developmental issues among young people in Hong Kong, such as growing adolescent substance abuse and worsening of young people's mental health. It is estimated that around 20% of young people in Hong Kong have psychosocial and adjustment problems in different domains[1,2]. Research findings also show that young people in Hong Kong are quite politically apathetic and they do not have a good understanding of Hong Kong society[3].

Second, research findings suggest that poor mental health among university students is an issue deserving our attention. For example, Wong et al.[4] found that there were high rates of psychological morbidity in first-year tertiary education students in Hong Kong. The Hong Kong Polytechnic University[5] reported that 10.1 and 5.9% of the students were classified as "Need Attention" and "Need Further Exploration", respectively. The Hong Kong Tertiary Institutions Health Care Working Group[6] showed that roughly half of the respondents displayed excessive anxiety problems. Shek[7] revealed that purpose in life in Chinese university students was comparatively poorer than in Western students.

Third, findings based on employer surveys commonly showed that employers in Hong Kong were not satisfied with the personal qualities of graduates in the areas of interpersonal cooperation and maturity. University students in Hong Kong also perceived that university students in mainland China performed better than they did in terms of sense of responsibility, problem solving abilities, interpersonal relationships, leadership, integrity, and public morality[8].

Fourth, although schools in Hong Kong commonly claim to emphasize "whole person" development, this emphasis is not reflected in the formal curriculum. For example, life skills training for secondary school students based on the curricular approach is not well developed in Hong Kong[9]. Similarly, although "whole person development" and "personal development" are commonly mentioned in the mission and vision statements of different universities in Hong Kong, attention is usually given to the intellectual development of university students (e.g., focus on Western and Chinese cultures) and universities have only paid lip service to holistic development of students. Thus, it is important to ask how university education can contribute to the holistic development of university students, particularly in the areas of psychosocial development and civic responsibilities.

Finally, it is noteworthy that different advisory bodies of the Government of the Hong Kong Special Administrative Region are actively emphasizing the importance of nurturing qualities in young people: the Family Council promotes core family values in Hong Kong people; the Committee on the Promotion of Civic Education promotes qualities of citizens in Hong Kong; and the Commission on Youth promotes qualities of young people through the Youth Charter. Against this background, one important question to resolve is how university education can contribute to the development of youth qualities promoted by these high-level Government advisory bodies.

### CONCEPTIONS AND MODELS OF HOLISTIC YOUTH DEVELOPMENT

Holistic youth development usually refers to the *total development* of an adolescent. While the idea of "total" may differ in different conceptual models, it is commonly agreed that holistic development includes physical, psychological, social, and spiritual domains. With particular reference to spirituality, it includes meaning of life, values, morality, forgiveness, and existential qualities, such as hope and forgiveness, which can be assessed by standardized rapid assessment tools[10].

Utilizing the framework of social-emotional learning in education, it is argued that in addition to focus on the academic achievement of students, one should also pay attention to the social and emotional development of young people. According to the Collaborative for Academic, Social and Emotional Learning (CASEL - http://www.casel.org), social and emotional learning (SEL) is "the process of acquiring the skills to recognize and manage emotions, develop caring and concern for others, make responsible decisions, establish positive relationships, and handle challenging situations effectively. Research has shown that SEL is fundamental to children's social and emotional development - their health, ethical development, citizenship, academic learning, and motivation to achieve. Social and emotional education is a unifying concept for organizing and coordinating school-based programming that focuses on positive youth development, health promotion, prevention of problem behaviors, and student engagement in learning." Generally speaking, several domains in SEL are identified, including self-awareness (e.g., identifying emotions and recognizing strengths), social awareness (e.g., perspective taking and appreciating diversity), self-management (e.g., managing emotions and goal setting), responsible decision making (e.g., analyzing situations, assuming personal responsibility, respecting others, problem solving), and relationship skills (e.g., communication, building relationships, negotiation, refusal). The above components of SEL are usually included in the conception of holistic development in young people.

Closely related to SEL is the field of positive development that focuses on each child's talents, strengths, interests, and future potential, in contrast to approaches that focus on some youth developmental problems, such as substance abuse. For example, Catalano et al.[11] found that positive youth development constructs are intrinsic to those successful positive youth development programs, including bonding, resilience, competencies (social, emotional, cognitive, behavioral, and moral competencies), self-determination, self-efficacy, spirituality, belief in the future, clear and positive identity, prosocial involvement, acquisition of prosocial norms, and recognition for positive behavior. There are validated instruments that assess positive youth development in the Western and Chinese cultures[12].

To formulate a strategy for the promotion of holistic development in university students in Hong Kong, we have to take into account some of the existing models of youth development and university education. There are theoretical models that focus on the importance of promoting adolescent developmental assets. For example, Benson[13] proposed 40 developmental assets for adolescents, which include commitment to learning, positive values, social competencies, and positive identity. Many researchers suggest that building cognitive, academic, social, and emotional competence is a fundamental task in adolescence. With reference to the specific assets to be developed, Weissberg and O'Brien[14] proposed five core social-emotional competencies to be targeted in positive youth development programs: self-awareness, social awareness, self-management, relationship skills, and responsible actions.

In their reflections on the work regarding positive youth development, Pittman et al.[15] explicitly argued that: (a) "problem free is not fully prepared"; (b) while academic competence is critical, it is not enough; (c) competence alone, while critical, is not enough; (d) development of four "Cs" (competence, confidence, character, connectedness) in young people are important; (e) three "Ls" (learning to be productive, learning to connect, learning to navigate) are important for youth development. Gardner[16] also argued that it is important for young people to develop the following attributes: the disciplined mind, the synthesizing mind, the creating mind, the respectful mind, and the ethical mind.

With specific reference to university education, Bok[17] argued that universities should help students to develop the following attributes: ability to communicate, critical thinking, moral reasoning, preparation for citizenship, living with diversity, living in a global society, development of a breadth of interests, and preparation for work. According to the Council for the Advancement of Standards for Higher Education[18], the following developmental outcomes are important for nurturing the leadership qualities of university students: intellectual growth, effective communication, enhanced self-esteem, realistic self-appraisal, clarified values, career choices, leadership development, healthy behavior, meaningful interpersonal relationships, independence, collaboration, social responsibility, satisfying and productive lifestyles, appreciating diversity, spiritual awareness, and personal and educational goals.

Other views, however, suggest that higher education in the contemporary world ignores holistic development of university students. In a special issue on the inner lives of university students, Dalton and Crosby[19] pointed out that higher education pays very little attention to the inner lives of university students and that "educational and student development efforts that ignore students' spirituality, i.e., how they make internal connections to the defining beliefs and purposes in their lives, will inevitably be less effective since they do not reach that part of students' lives where things really matter" (p. 1). Dalton and Crosby[20] further argued that "the increasing emphasis on making money as a primary measure of success reflects a shift in college students' focus on the achievements of one's inner life and character to the external accumulation of money and the amenities and status associated with wealth" (i.e., a shift from "being" to "having"). They pointed out that in contrast to colleges a century ago that focused much on "internal" measures of excellence, such as values, character, integrity, and responsibility, higher education in America has neglected the inner lives of college students and focuses on "external" measures of success, such as financial success. Based on the above views, the primary mission of university education is not as simple as impartation of knowledge.

### HOW DO WE PROMOTE HOLISTIC DEVELOPMENT IN UNIVERSITY STUDENTS?

Obviously, there are different ways of nurturing holistic development in university students. For example, we can develop a more accepting and empowering campus environment so that students can reflect more on their inner lives. Historically, the Student Affairs Office (SAO) is charged with the mission to promote holistic development in university students and there is no exception in Hong Kong. The reliance on SAOs to undertake this job may be due to two factors. First, SAOs normally have experts, such as clinical psychologists, to deal with mental health and adjustment issues among university students. Actually, it is a usual practice for universities in Hong Kong to employ clinical psychologists and workers who specialize in counseling psychology in SAOs. Second, unlike guidance teachers in high schools, teachers in academic departments are usually not trained to manage students' personal problems. Even in teaching departments where colleagues have training in social science, such as psychology and social work, they may also find it difficult, if not impossible, to tackle student developmental issues.

While SAOs have the expertise to deal with student issues, there are several limitations of solely relying on SAOs to promote positive development among university students. First, the student counseling service in SAOs is commonly operated on a "clinical" model, waiting for the visits of the students. This is not a desirable intervention model as students only seek help at a very late stage of their problems and they have low readiness to seek help. Second, the "clinical" model has a strong flavor of overemphasis on the deficits of the students (i.e., pathological model), although SAOs also provide programs based on theories and concepts on positive psychology. Third, while primary prevention programs are offered by SAOs, students normally participate in these programs in a voluntary manner outside class time. This means that some needy students would be "left behind".

An alternative and nonmutually exclusive approach to promote holistic youth development is to develop and provide curricula-based courses on holistic youth development. In North America, there is a strong emphasis on social and emotional learning in high school students and curricula-based positive youth development programs are commonly implemented. Interestingly, such programs begin to disappear in university settings. There are several assumptions related to this phenomenon. First, there is the assumption that university students are grown-up adults and there is no need to teach them how to handle their lives. Second, there is the assumption that students should focus on "academic" subjects, and courses on personal development and psychosocial competencies are relatively unimportant. Third, there is the assumption that students should be given the option to take courses on holistic development and any required course is inappropriate.

To what extent are the above assumptions valid? Regarding the first assumption, the literature review clearly shows that university students display many problems, such as mental health problems[4]. For the second assumption, it is basically a matter of academic vision. For example, universities normally have compulsory language courses because universities regard languages as basic to learning. Some universities also have required courses on Western and Chinese cultures because they believe that such training can broaden the cultural perspectives of the students. By the same token, it can be argued that psychosocial competencies should be regarded as vital and basic if we believe that holistic development of university students is important.

Finally, the question of whether students should be given the option to enroll in courses on holistic development depends on our educational vision and perspectives. Why do we require university students to take languages and culture courses, but not holistic development courses? With reference to the question of "what is the most important course in a university?", Crosby[21] argued that "a major task of the university, at least one that seeks to operate in the tradition of the liberal arts, is to help students find their moral and spiritual bearings and to assist them in the critical task of orienting themselves in the world" and warned that "the search for a holistic vision of life is left in the typical modern university largely to students and is more a topic of dormitory bull sessions than of formal class settings" (p. 3). Definitely, universities should promote "freedom from ignorance" about oneself including one's inner life.

### PROMOTION OF HOLISTIC DEVELOPMENT VIA CREDIT-BEARING COURSES

With reference to university education, it is noteworthy that freshman seminars and general education courses are rarely developed to promote holistic youth development. With a few exceptions, general education programs in different universities in Hong Kong are also relatively similar. The common mode adopted is that there are some compulsory courses (mainly on languages and cultures) plus selection of some courses from different domains (e.g., science and technology, social science, and arts and humanities domains). Obviously, it can be criticized that such a "buffet" style of general education program design lacks vision and it cannot adequately respond to the needs of university students[21,22]. There are growing criticisms about this "distribution approach" that fails to help university students develop qualities with personal relevance. Another sad observation is that the design of general education courses seldom takes into account the literature on adolescent developmental psychology, psychiatry, youth work, and youth participation in the community.

We can further criticize that university general education programs in Hong Kong are geared towards survival (i.e., survival education that attempts to increase the chance of the graduates to find jobs), but not "living" education (i.e., helping students learn how to live by helping them to develop life skills) and life education (i.e., helping students to reflect on life and find a purpose). The failure of university education in nurturing university students in a holistic manner is highlighted in the literature[17,22].

In the process of transforming to the 3-3-4 curriculum (3 years of junior secondary, 3 years of senior secondary, and 4 years of university), universities in Hong Kong have commonly put more focus on general education and freshman seminar courses. Against the above background, it is argued that several principles should be seriously considered in the design of general education or freshman seminar courses to promote holistic development in university students in Hong Kong:

1. ***Holistic student development*:** It is important to respond to the *total needs* of the students. The focus should be authentic holistic development that is carried out in an honest and whole-hearted manner.
2. ***Responding to community concern about young people*:** University general education courses should help to nurture students with respect to the community concern that young people lack social and emotional competencies and moral values. General education courses should also respond to the Government's expectation that universities in Hong Kong should nurture young people in terms of civic responsibilities and citizenship.
3. ***Preparing students for adulthood and general education with life-long benefits*:** It is argued that transition to adulthood in contemporary society is not automatic. Karen Pittman's assertion that "problem free is not fully prepared" clearly suggests that preparation for adulthood is important[15]. As such, we should develop some general education courses that can help students in their university years as well as having life-long benefits for the students. As Lewis[22] pointed out, we should rethink whether we can "help [our students] to grow up, to learn who they are, to search for a larger purpose of their lives, and to leave college as better human beings" (p. xii).
4. ***Uniqueness*:** The general education program should be visionary and unique, which can explicitly communicate the message to society that we are concerned about the holistic development of students.
5. ***Universal coverage*:** We must make sure that every student can develop basic personal and leadership qualities that have applied relevance to their lives. Students who take the courses should also learn to develop qualities regarded as important by the community.

6. ***Theory-driven general education program*****:** The design of basic general education courses that target personal and leadership qualities must be based on well-articulated theoretical models. As mentioned above, numerous adolescent developmental models and frameworks on university education emphasize the importance of developing psychosocial competencies, civic responsibilities, and citizenship in young people.
7. ***Research-driven general education program*****:** The design of basic general education courses on personal and leadership qualities, as well as civic responsibilities, must also be based on research findings. Previous studies have shown that personal and leadership qualities contribute to the overall development of young people[23].

Obviously, curricular-based holistic education and other youth enhancement initiatives, such as those provided by the SAOs, and development of a health-promoting campus environment are not mutually exclusive. However, from a public health perspective, it is important to know whether *each* student has the opportunity to acquire the related knowledge and skills (i.e., freedom from ignorance about oneself).

### LEADERSHIP DEVELOPMENT AS A CONCEPTUAL FRAMEWORK FOR HOLISTIC YOUTH DEVELOPMENT

To nurture young people in a holistic manner, it is proposed that leadership courses should be designed for university students. A review of the literature shows that there are numerous models of leadership. For example, Yammarino et al.[24] conducted a comprehensive review of leadership models with reference to different levels of analysis and issues. Four major categories of leadership approach comprising 17 leadership models were identified: (1) classical approach (the Ohio State Model, the Contingency Model, Participative Leadership Model, Situational Ladership Model, Path-Goal Theory, and Vertical Dyad Linkage Model); (2) contemporary approach (Charismatic Leadership Model, Transformational Leadership Model, and Leader-Member Exchange Model); (3) alternative approach (Information Processing and Implicit Theories Model, Substitutes for Leadership Model, Romance of Leadership Model, and Influential Tactics Model); and (4) new wave approach (Self-Leadership Model, Multiple Linkage Model, Multilevel and Leaderplex Models, and Individualized Leadership Model). In short, it is noteworthy that different models of leadership can be found in the literature and, interestingly, many models that emphasize the importance of certain qualities of a leader are conducive to successful leadership. Some examples are outlined in the following paragraphs.

#### Developmental Leadership Model[25,26]

The Developmental Leadership Model states that leadership behaviors are developed by the dynamic interplay between leader and contextual characteristics. It is proposed that two domains of leader characteristics (basic prerequisites and desirable competencies) interact with three aspects of contextual characteristics (the environment, organization, and group) to develop different leadership styles. For the desirable competencies, they include task-related competence, management-related competence, social competence, and capacity to cope with stress. For basic prerequisites, they include physical, psychological, and view-of-life-related attributes. In short, one argument of this model is that it is important to cultivate leadership attributes in terms of the basic attributes and desirable competencies.

#### Transformational Leadership Model[27,28]

The Transformational Leadership Model emphasizes that leaders exert influence on followers through underlying motivational processes so as to raise the followers' levels of awareness beyond their selfinterest for the enhancement of the collective interests in the group, organization, and society at large. A leader under this approach is expected to foster a sense of trust, loyalty, and respect in the followers. Four key components are found as motivating factors in this model, including charisma or idealized influence, inspirational motivation, intellectual stimulation, and individualized consideration. Again, specific qualities are highlighted in successful leaders.

#### Competency-Based and Trait-Based Leadership Models[29,30]

According to Brownell[29], certain competencies (including common competencies and distinctive competencies) contribute to high-performing global leaders. Several distinctive competency clusters, including intercultural, social, creativity/resourcefulness, self-knowledge, positive outlook, responsiveness, and decision making, are proposed. In particular, character, trustworthiness and integrity are strongly emphasized in the model. In the model proposed by Zaccaro et al.[30], it was proposed that distal attributes (cognitive abilities, personality, and motives values) and proximal attributes (social appraisal skills, problem-solving skills, and expertise/tacit knowledge) determine leadership processes. In their study of leadership excellence in ASEAN nations, Taormina and Selvarajah[31] found four important qualities of leaders, including considerations for others, progressive stability, strategic thinking, and trust in others. One important implication of these models is that the basic qualities of leaders should be nurtured in leadership training programs, particular qualities related to inner lives, interpersonal communication, and knowledge about the greater social milieu in which one is situated.

### PROPOSED COURSES ON LEADERSHIP TRAINING

Adopting the philosophy of "leadership for all" and "everyone is Number 1", two courses are proposed to help university students develop their leadership qualities and skills. The subject proposals can be seen in Appendix 1 and Appendix 2.

The first proposed course is entitled "Tomorrow's Leaders I". The course is designed to enable students to learn and integrate theories, research, and concepts on the basic personal qualities (particularly intra- and interpersonal qualities) of effective leaders within the Chinese context. This subject also intends to train students to develop and reflect on their intra- and interpersonal qualities. Finally, the course promotes the development of an active pursuit of knowledge on personal qualities in leadership among students. Of course, being a leader in some areas does not mean that one is a leader in all areas. Learning to be a productive, contributing follower is definitely important.

The second proposed course is entitled "Tomorrow's Leaders II". For an effective leader, he/she should know his/her community and country. The course is designed to enable students to learn and integrate knowledge, research, and concepts on civic responsibilities and citizenship, with particular reference to university students and graduates within the Chinese context. This subject also intends to familiarize students with the cultural, social, and political characteristics of Hong Kong and China. Finally, students are helped to reflect on how university students and university graduates can contribute to the development of Hong Kong and China.

There are several arguments for adopting these two courses as compulsory for university students in Hong Kong. First, research findings show that young people in Hong Kong lack intra- and interpersonal skills. Second, research findings indicate that young people in Hong Kong lack understanding of Hong Kong (i.e., the community that they are living in), China (i.e., young people's mother country), civic responsibilities, and citizenship. Third, findings based on employer surveys commonly show that employers in Hong Kong are not satisfied with the personal qualities of graduates in the areas of interpersonal cooperation and personal maturity. Fourth, many theorists, researchers, and educators criticize that universities in the contemporary world fail to help students to develop the following attributes: ability to communicate, critical thinking, moral reasoning, preparation for citizenship, living with diversity, living in a global society, development of a breadth of interests, and preparation for work. We need freshman seminar and general education courses to respond to such a criticism. Fifth, the proposed courses are constructive responses to the Government's expectation that young people should have a high sense of civic responsibilities and citizenship. Sixth, no university in Hong Kong requires undergraduate students to take courses on intra- and interpersonal development, civic responsibilities and citizenship, Hong Kong society, and China.

Obviously, several issues should be addressed when these two courses are developed as required courses for university students. First, we need to train teachers to teach these courses. Ideally, teachers with a social science background are strongly preferred to teach these courses. Second, monitoring of the quality of the courses is also important if the courses are offered on a massive scale. Finally, systematic evaluation that monitors the effectiveness of these courses should be carried out. Similar to positive youth development programs designed for high school students, such as the Project P.A.T.H.S.[32,33,34,35], these tasks can form an interesting research program that will have impact not just for Hong Kong, but also for China and the global world.

## APPENDIX 1

### Course on "Tomorrow's Leaders I": Specific Objectives of the Subject

The course is designed to enable students to learn and integrate theories, research, and concepts of the basic personal qualities (particularly intra- and interpersonal qualities) of effective leaders within the Chinese context. This subject also intends to train students to develop and reflect on their intra- and interpersonal qualities. Finally, the course promotes the development of an active pursuit of knowledge on personal qualities in leadership among students. Upon completion of the subject, students will be able to:

1. Understand and integrate theories, research, and concepts on the basic qualities (particularly intra- and interpersonal qualities) of effective leaders in the Chinese context
2. Develop self-awareness and understanding of oneself
3. Acquire interpersonal skills
4. Develop self-reflection skills in their learning
5. Recognize the importance of active pursuit of knowledge on intra- and interpersonal leadership qualities

#### *Unique Features of the Course*

1. Literacy: Students will be required to read extensively, with reading materials covering a wide range of disciplines, including developmental psychology, positive psychology, organizational psychology, counseling, social work, communication, experiential learning. Generally speaking, students are required to read at least 20 pages of books and journal articles per week. Students are also required to write reflective journals and to submit a project report with at least 3,000 words.
2. High-order thinking: Students will be required to understand and integrate theories, concepts, and research on leadership qualities with reference to the Chinese culture. Development of self-reflection skills is also strongly emphasized in the course.
3. Life-long learning: Students are expected to develop independent learning habits and reflection about their learning styles in the course. In particular, the course attempts to promote the motivation of students in pursuing life-long learning. Students are expected to apply what they have learned to real life settings.

#### *Subject Synopsis*

1. An overview of the personal attributes of effective leaders: role of self-understanding and interpersonal relationship qualities
2. Self-understanding: theories and concepts; self-concept, self-esteem, and personal identity; the role of self-understanding in effective leadership
3. Emotional competence: awareness and understanding of emotions; emotional quotient (EQ); role of emotional management in effective leadership
4. Cognitive competence: different types of thinking; higher-order thinking; experiential learning; role of cognitive competence in effective leadership
5. Resilience: stresses faced by adolescents; life adversities; coping with life stresses; adversity quotient (AQ); role of resilience in effective leadership
6. Spirituality: meaning in life and adolescent development; spirituality and mental health; role of spirituality in effective leadership
7. Ethics and morality: moral issues and moral competence; role of ethics and morality in effective leadership

8. Social competence: basic social competence skills; ability to build up positive human relationship; role of social competence in effective leadership
9. Interpersonal communication: theories, skills, and blocks of interpersonal communication; role of communication skills in effective leadership
10. Interpersonal conflict: theories of interpersonal conflict; conflict resolution skills; role of conflict resolution in effective leadership
11. Relationship building and maintenance: tactics of building and maintaining relationship; relationship quality and effective leadership
12. Team building: tactics and strategies of team building; identifying common goals in a team; maintaining morale and dealing with demoralization
13. Assertiveness: respecting the views of oneself and others; feelings of guilt related to assertiveness and nonassertiveness; being an assertive leader
14. Interpersonal harmony: nature of interpersonal harmony; interpersonal harmony in the Chinese culture; role of interpersonal harmony in effective leadership

### *Teaching and Learning Methodology*

Students who enroll in this course are expected to be sensitive to their own behavior in the intra- and interpersonal contexts. Both intellectual thinking and experiential learning are emphasized in the course. The teaching/learning methodology includes:

1. Lectures
2. Tutorial and experimental learning sessions
3. Group project
4. Written assignments (reflection journals and project report)

### APPENDIX 2

### Course on "Tomorrow's Leaders II": Specific Objectives of the Subject

For an effective leader, he/she should know his/her community and country. The course is designed to enable students to learn and integrate knowledge, research, and concepts on civic responsibilities and citizenship, particularly with reference to university students and graduates within the Chinese context. This subject also intends to familiarize students with the cultural, social, and political characteristics of Hong Kong and China. Finally, students are expected to reflect on how university students and university graduates can contribute to the development of Hong Kong and China. Upon completion of the subject, students will be able to:

1. Understand and integrate theories, research, and concepts of civic responsibilities, citizenship, and contributions of intellectuals to social development
2. Understand the cultural, social, and political characteristics, as well as social needs of Hong Kong
3. Familiarize oneself with the cultural, social, and political characteristics, as well as social needs of China
4. Develop self-reflection about one's civic responsibilities and citizenship
5. Recognize the importance of active pursuit of knowledge on civic responsibilities and citizenship, and develop related action plans

#### *Unique Features of the Course*

1. Literacy: Students will be required to read extensively, with reading materials covering a wide range of disciplines, including sociology, social policy, social work, social philosophy, political science, youth development, and experiential learning. Generally speaking, students are required to read at least 20 pages of books and journal articles per week. Students are also required to write reflective journals and to submit a term paper with at least 4,000 words.
2. High-order thinking: Students will be required to understand and integrate theories, concepts, and research on civic responsibilities and citizenship with particular reference to Chinese university students and graduates. Development of self-reflection skills is also strongly emphasized in the course.
3. Life-long learning: Students are expected to develop independent learning habits and reflection about their learning styles in the course. In particular, the course attempts to cultivate the motivation of the students in pursuing life-long learning. Students are expected to apply what they have learned to real-life settings, particularly on how they contribute to the development of Hong Kong and China.

#### *Subject Synopsis*

1. An overview of the personal attributes of effective leaders: understanding of and concern for one's society and country; civic responsibilities and citizenship
2. Citizenship and civic responsibilities: theories, concepts, and research
3. Roles and responsibilities of university students: community service; student movements in China and overseas
4. Roles and responsibilities of professionals: professional values and ethics; contribution of professionals to social development
5. Citizenship and civic responsibilities: basic concepts and ways to promote citizenship and civic responsibilities in university students; possible action plans

6. Understanding Hong Kong society: historical overview and transition from a British colony to a Special Administrative Region
7. Major social needs and problems in Hong Kong: phenomena, perspectives, and possible solutions
8. Social development in Hong Kong: the Social Development Index (SDI); vulnerable groups in Hong Kong
9. Political participation and social involvement of young people in Hong Kong: promoting political participation of university students in Hong Kong
10. Understanding contemporary China: historical review and the Open Door Policy in the past 3 decades
11. Major social needs and problems in mainland China: phenomena, perspectives, and possible solutions
12. Social development in China: the Human Development Index; vulnerable groups in China; emerging social issues
13. Political participation and social involvement of young people in China: phenomena, perspectives, and issues
14. Contribution of university students and graduates to the development of Hong Kong and China

### *Teaching and Learning Methodology*

Students who enroll in this course are expected to be sensitive to their civic responsibilities and possible contribution to the development of Hong Kong and China. Both intellectual and experiential learning are emphasized in the course.

1. Lectures
2. Tutorials and community experience project
3. Group project
4. Written assignments (reflection journals and project report)
